# A favorable lifestyle lowers the risk of coronary artery disease consistently across strata of non-modifiable risk factors in a population-based cohort

**DOI:** 10.1186/s12889-019-7948-x

**Published:** 2019-11-27

**Authors:** Kristian Dimovski, Marju Orho-Melander, Isabel Drake

**Affiliations:** 1 Diabetes and cardiovascular disease – genetic epidemiology, Department of Clinical Sciences in Malmö, Lund University, Lund, Sweden; 2 Clinical Research Centre, Jan Waldenströms gata 35, 205 02 Malmö, Sweden

**Keywords:** Coronary artery disease, Lifestyle, Cohort study, Relative risk, Cumulative risk

## Abstract

**Background:**

A healthy lifestyle has been shown to reduce the risk of coronary artery disease (CAD). The extent to which lifestyle influences the risk of CAD for people with pre-existing non-modifiable risk factors is less studied. We therefore examined the associations between a favorable lifestyle and incidence of CAD in population subgroups based on gender, age, educational level, and parental history of myocardial infarction.

**Methods:**

A total of 26,323 men and women from the Malmö Diet and Cancer study were prospectively followed-up for 18 years. A favorable lifestyle was determined using a four-component lifestyle score based on data collected at baseline: no smoking, no obesity, regular physical activity, and a healthy diet. Cox proportional hazards regression models were used to estimate the relative risk of CAD during follow-up and cumulative risk during a 10-year interval.

**Results:**

A favorable lifestyle was associated with a 44% (95% confidence interval, 38–48%) lower risk of CAD compared to an unfavorable lifestyle. The relative risk was similarly reduced among subjects subdivided by gender, age group, educational level, and parental history of myocardial infarction. These findings corresponded with a reduced standardized 10-year incidence of CAD of around 40% in each subgroup.

**Conclusion:**

In this population-based cohort, a favorable lifestyle was associated with a significant reduction of CAD across strata of non-modifiable risk factors. These findings provide support for lifestyle modification as a means for risk reduction in a range of subgroups within a general healthy population.

## Background

There is strong evidence that reduction in cardiovascular disease (CVD) risk is feasible through both the individual and cumulative adherence to healthy lifestyle choices including abstinence from smoking, regular physical activity, avoiding obesity and consuming a healthy diet [1–5]. Promoting adherence to a healthy lifestyle in the population therefore constitutes an important strategy for lowering CVD risk. The impact of lifestyle modification in population subgroups with concurrent, non-modifiable risk factors is less studied and consequently risk communication and the priorities for these population subgroups are less clear. There are a number of non-modifiable risk factors for CVD. Age over 65 years and male gender are the two strongest established CVD risk factors [6, 7]. The association between parental history of myocardial infarction (MI) and future cardiovascular events has been shown to be constant regardless of age, gender, geographical living area and socioeconomic background [8, 9]. Therefore, it provides a simple and effective clinical assessment of CVD risk. Likewise, people with a lower educational level have a higher risk of developing CVD potentially by having a more unfavorable pattern when it comes to CVD risk factors such as smoking and overweight [10, 11]. Large observational studies of healthy populations that have examined the impact of lifestyle or individual lifestyle factors on subsequent CVD risk have adjusted for non-modifiable risk factors. A recent study showed that both genetic and lifestyle factors are independent variables associated with increased risk for coronary artery disease (CAD) [6]. Among participants with high genetic risk, a favorable lifestyle was associated with a 50% lower risk of CAD compared to those with an unfavorable lifestyle [12]. However, few studies have specifically examined whether other non-modifiable risk factors may modify the benefit of adherence to a favorable lifestyle. The main purpose of this study was therefore to analyze the potential CAD risk reduction by adherence to a favorable lifestyle within population subgroups based on gender, age, educational level and parental history of MI.

## Methods

### Study population

The Malmö Diet and Cancer study (MDCS) is a prospective population-based cohort study that was designed to study the association between diet and other lifestyle factors on the risk of developing cancer [13]. Both male and female participants between the age of 43 and 73 years in Malmö took part in the study, with baseline examinations from March 1991 to October 1996 [14]. From the cohort of 30,446 subjects, 923 subjects with a previous CVD (coronary event or stroke) identified through local or national registers were excluded as well as 150 with history of coronary artery bypass grafting (CABG) or percutaneous coronary intervention (PCI). Subjects with missing data on the main covariates (1185 with missing lifestyle score, 6 with missing age or sex and 1859 with missing educational level) were also excluded. The total study population after exclusions was 26,323.

### Endpoint ascertainment

CAD was defined as fatal or nonfatal MI, death due to ischemic heart disease, PCI, or CABG, whichever came first. Time of follow-up was counted from the date of baseline examination until first event, death, emigration or 31st December 2014. End-point adjudication was done through linkage of the personal identification number with three registers: the Swedish Hospital Discharge register, the Swedish Cause of Death Register, and the Swedish Coronary Angiography and Angioplasty Registry.

### Lifestyle score

A lifestyle score was constructed using four lifestyle factors in the American Heart Association (AHA) guidelines – no current smoking, no obesity (BMI < 30 kg/m^2^), moderate physical activity at least once weekly, and a healthy diet. These guidelines are similar to lifestyle recommendations issued for disease prevention in Sweden. A favorable lifestyle was defined as adherence to 3 or more of the listed criteria, an intermediate lifestyle as two criteria and an unfavorable lifestyle as adherence to 1 or zero of the criteria, as previously reported [12]. Information on smoking status and physical activity was extracted from the baseline questionnaire. Non-smoking was defined as reporting never having smoked or being a former cigarette smoker. Physical activity was based on participants reporting the number of minutes per week spent on a list of 18 activities during the four seasons (adapted from the Minnesota Leisure Time Physical Activity Instrument). Physical activity at least once weekly was defined as reporting more than 1-h of activities with a proximal intensity of at least 5 METs (corresponding to moderate/vigorous intensity). The activities included digging, dancing (folkdance and ballroom), mowing the lawn (manual mower), jogging, swimming, tennis, soccer, orienteering, and climbing stairs. Absence of obesity (BMI < 30 kg/m^2^) was defined by calculated BMI from direct measurements of height and weight by trained nurses using standardized procedures. Dietary assessment in the MDCS was by a modified diet history method combining 1) a 7-day diet record of all prepared meals, cold beverages, and supplements, 2) a 168-item quantitative food questionnaire using pictures to assess portion sizes, and 3) a 1-h interview with a trained interviewer to check for accuracy of the diet record and food questionnaire, potential overlap, and general food habits and cooking practices [15]. Information from the diet record and food questionnaire were used to estimate g/day of different food items. The relative validity and reproducibility of the method have been published [16–18]. A healthy diet pattern was calculated based on adherence to at least 5 of the following criteria: consumption of more fruits, nuts, vegetables, whole grains, fish, and dairy products, and less consumption of refined grains, processed meats, unprocessed red meats, and sugar-sweetened beverages. A detailed description of the included foods in the respective food groups and serving sizes used for assessing adherence is found in Additional file 1: Table S1.

### Non-modifiable risk factors and other covariates

Population subgroups were classified based on the following non-modifiable risk factors: age, gender, educational level, and parental history of MI. Age and sex were extracted from the subjects’ Swedish personal identification number. The participants were divided into three age groups: 43.0–54.9 years, 55.0–64.9 years, and 65.0–73.9 years with the oldest age group being defined as high-risk. Information on educational level and parental history of MI was extracted from the baseline questionnaire. Educational level was categorized in three categories: elementary, secondary, or further education/university/college degree. Elementary degree was defined as a high-risk group. Parental history of MI was determined by the participants answer on whether they have a father and/or mother with a history of myocardial infarction. For descriptive analyses of the cohort across exposure categories we further examined history of hypertension (based on blood pressure measurements above 140/90 mmHg or reported use of anti-hypertensive medication at baseline), use of lipid-lowering medication in the MDC baseline questionnaire, and history of diabetes mellitus was defined as any of the following: having a measured fasting whole blood glucose ≥6.1 mmol/l at the MDC baseline examination, or a self-reported history of physician-diagnosed diabetes, or use of diabetes medication according to the MDC baseline questionnaire, or being diagnosed and registered in any of the local or national diabetes registries. Further, in a subset of participants (*n* = 4995) fasting blood samples were collected at baseline as previously described [19]. For these subjects, we included analysis of baseline measurements of HbA1c (%), triglycerides (mmol/L), and high-density lipoprotein cholesterol (HDLC; mmol/L). Low-density lipoprotein cholesterol (LDLC) was estimated using Friedewald’s formula. Analyses of high-sensitivity C-reactive protein (hsCRP) was performed using the Tina-quant® CRP latex high sensitivity assay (Roche Diagnostics, Basel, Switzerland) on an ADIVA® 1650 Chemistry System (Bayer Healthcare, NY, USA).

### Statistical analyses

We examined baseline characteristics across categories of lifestyle score (favorable, intermediate, unfavorable). Continuous variables across lifestyle categories are expressed as mean values with standard deviation and differences were tested using ANOVA. Skewed continuous variables are expressed as median with interquartile range and differences were tested using Kruskal-Wallis ranksum test. The distribution of subjects across categorical variables are expressed as frequency (%) and differences in distribution was tested using the chi-square test. Baseline characteristics were further examined for each of the population risk groups: age, gender, educational level, and parental history of MI. Cox proportional hazards regression models were used to model the association between the lifestyle score and incident CAD overall and stratified by non-modifiable risk factors. Hazard ratios (HR) with 95% confidence intervals (CI) were estimated and adjusted for age, sex, educational level and parental history of MI. The proportional hazards assumption was tested using the Schoenfeld residuals; no deviation was noted. The cumulative risk (standardized 10-year coronary event rate based on a Cox regression model) by lifestyle categories was further estimated within strata of non-modifiable risk factors. All analyses were performed in Stata/SE (Version 14.2, College Station, TX, USA) and R (Version 3.2.1). All tests were two-sided and *P*-values < 0.05 were considered as statistically significant.

## Results

### Description of the study population

With a mean follow-up time of 18 years (median 19.6, range 0–23.8) there was in total 469,441 person-years of observation during which 3417 incident events of CAD occurred. A favorable lifestyle was associated with a better risk profile in relation to known CAD risk factors (Table 1). Participants with a favorable lifestyle had a lower likelihood of hypertension and favorable blood-lipid levels as well as being less likely to have diabetes at baseline. Baseline characteristics across the population risk groups (age, gender, educational level, and parental history of MI) are shown in Additional file 1: Table S2-S5.

**Table 1:** Baseline characteristics across categories of a lifestyle score in the Malmö Diet and Cancer Study (1991–1996)

	Unfavorable Lifestyle *N* = 5730	Intermediate Lifestyle *N* = 11,757	Favorable Lifestyle *N* = 8846	P-value**
Age, years^	57.3 (7.4)	58.0 (7.7)	58.1 (7.6)	< 0.0001
Gender ¤				< 0.0001
Male, %	34.6	36.6	42.4	
Female, %	65.4	63.4	57.6	
Education ¤				< 0.0001
Elementary %	48.2	41.2	35.9	
Secondary %	33.8	36.1	35.8	
University/college degree, %	17.9	22.6	28.3	
Parental history of MI, % ¤	31.2	32.6	31.7	0.12
History of Hypertension ¤	64.2	60.0	58.3	< 0.0001
History of Diabetes Mellitus ¤	5.2	3.5	3.8	< 0.0001
Body-mass Index, kg/m^2^ ^	27.3 (5.2)	25.4 (3.6)	25.0 (2.9)	< 0.0001
Lipid Levels*				
LDL Cholesterol, mg/dl^	4.24 (1.03)	4.16 (0.99)	4.14 (0.97)	0.034
HDL Cholesterol, mg/dl^	1.30 (0.34)	1.40 0.38)	1.42 0.37)	< 0.0001
Triglycerides, mg/dl #	1.31 (0.97–1.82)	1.15 (0.87–1.59)	1.09 (0.82–1.51)	0.0001
Lipid-lowering Medication, % ¤	2.3	2.2	2.1	0.67
C-Reactive Protein* #	1.9 (0.9–4.2)	1.3 (0.7–2.7)	1.1 (0.6–2.2)	0.0001
HbA1C* #	4.9 (4.6–5.3)	4.8 (4.5–5.1)	4.7 (4.4–5.0)	0.23

* Only subjects from the MDC cardiovascular cohort (*N* = 4995)

^Continuous variables are expressed as mean values with standard deviation in parenthesis. Differences in mean values were tested using one-way ANOVA

#Skewed continuous values are expressed as median with interquartile range in parenthesis. Differences in median were tested using Kruskal-Wallis ranksum test

¤ The distribution of subjects across the categorical variables are expressed as frequency (%). The difference in distribution was tested using the chi-square test

** *P*-values < 0.05 were considered as statistically significant

### Lifestyle, non-modifiable risk factors and risk of CAD

The CAD event rates over study follow-up across categories of lifestyle risk and different population risk groups are shown in Additional file 1: Fig. S1. Participants with an unfavorable lifestyle had a higher risk of CAD than those with a favorable lifestyle with a HR of 1.77 (95% CI: 1.62–1.94). Similarly, the adjusted event rate was higher among all high-risk groups compared to low-risk groups (Additional file 1: Fig. S1).

**Fig. 1:**
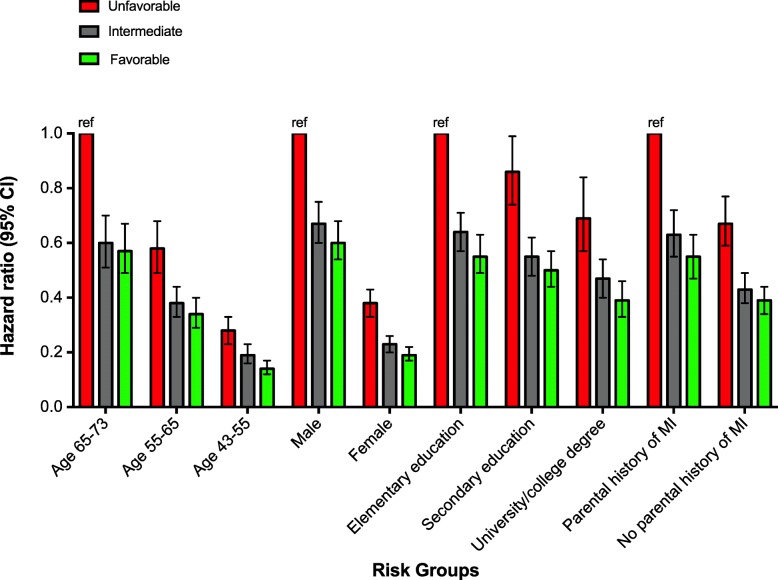
Hazard ratios (HRs) and 95% confidence intervals (CIs) from Cox proportional hazard model by lifestyle risk score and categories of age, gender, educational level, parental history of MI, and ASCVD risk score. Unfavorable lifestyle and high-risk categories were used as reference categories (denoted ref). Models were adjusted for sex, age, educational level and parental history of MI

Across all strata of non-modifiable risk factors, adherence to a favorable lifestyle was associated with a lower risk of CAD (Fig. 1). Overall, a favorable lifestyle associated with a 44% lower risk of CAD (HR = 0.56, 95% CI: 0.52–0.62) compared to an unfavorable lifestyle. In the older age group (65–73 years), the participants who adhered to a favorable lifestyle had a 43% (HR = 0.57, 95% CI: 0.49–0.67) lower risk to develop CAD compared to participants at the same age with an unfavorable lifestyle. For men with a favorable lifestyle the relative risk was 40% lower (HR = 0.60, 95% CI: 0.54–0.68) compared to men with an unfavorable lifestyle. Participants with a low educational level reduced their relative risk with 45% (HR = 0.55, 95% CI: 0.49–0.63) by adhering to a favorable lifestyle. Likewise, compared to an unfavorable lifestyle subjects with a parental history of MI who adhered to a favorable lifestyle reduced their relative risk of CAD with 45% (HR = 0.55, 95% CI: 0.47–0.63) (Fig. 1 and Additional file 1: Table S6).

**Fig. 2:**
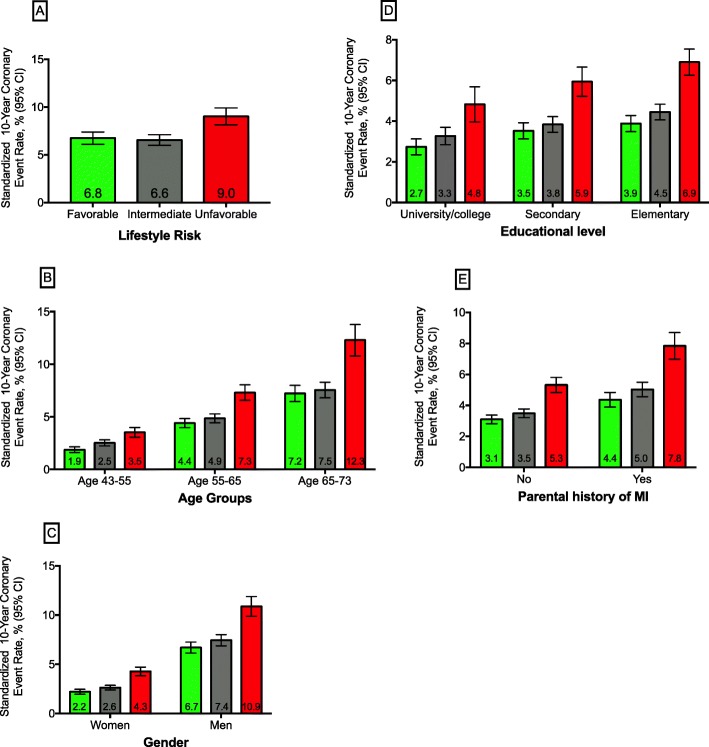
Standardized 10-year coronary event rate (%) by lifestyle categories (**a**) and stratified by age (**b**), gender (**c**), educational level (**d**), and parental history of MI (**e**)

For participants at high age the standardized 10-year coronary event rate was 12.3% (95% CI: 10.8–13.8) among those with unfavorable lifestyle and 7.2% (95% CI: 6.4–8.0) among those with a favorable lifestyle (Fig. 2). A similar pattern was noted in the other high risk groups where a favorable lifestyle lowered the absolute risk in these groups compared to an unfavorable lifestyle. For men with an unfavorable lifestyle the 10-year coronary event rate was 10.9% (95% CI: 9.9–11.9) compared to 6.7% (95% CI: 6.1–7.3) with a favorable lifestyle. The group with elementary education and an unfavorable lifestyle had a cumulative risk of 6.9% (95% CI: 6.3–7.5) which was reduced to 3.9% (95% CI: 3.5–4.3) with a favorable lifestyle. For participants with a history of MI, an unfavorable lifestyle was associated with a 7.8% (95% CI: 7.0–8.7) absolute risk of CAD compared to a 4.4% (95% CI: 3.9–4.8) with a favorable lifestyle.

## Discussion

In this study, we examined the putative effect of a favorable lifestyle across strata of established non-modifiable risk factors. Overall, we found that a favorable or intermediate lifestyle compared to an unfavorable lifestyle was associated with a significantly lowered risk of CAD regardless of the population subgroup. The risk reduction corresponded to a 10-year cumulative risk reduction of between 40 and 50% in each of the high-risk groups.

A meta-analysis including 22 studies examining the association between a combination of different lifestyle risk factors on risk of CVD was recently published [20]. While operationalized lifestyle scores differ, there was a consistent inverse association between a healthy lifestyle and CVD risk with a summary HR estimate of 0.34 (95% CI: 0.28–0.41). The results of the current study are thus in line with several previous studies [20]. A Swedish cohort study examined the impact of healthy lifestyle in a population with high-risk of stroke. Adherence to five healthy lifestyle factors compared to one or zero factors reduced the relative risk of stroke with 72% [21]. Another cohort study of 60-year-old men and women found that adherence to healthy lifestyle factors reduced the relative risk of CVD incidence and death, regardless of BMI and educational level [22]. Similarly, a population-based prospective cohort study in Sweden, examined the effect of five lifestyle factors (healthy diet, moderate alcohol consumption, physical activity, no smoking and no abdominal adiposity) on the incidence of MI in men [23]. The AHA Cardiovascular Health Index (CVHI) have previously been constructed and implemented [24]. Gooding et al. operationalized the four lifestyle components of the CVHI in relation to cardiovascular health in younger adults followed into middle-age. This study showed that a high adherence to the AHA recommendations associated with lower likelihood of progressing to poor cardiovascular health, suggesting benefit of early implementation of favorable lifestyles [25]. Within the Atherosclerosis Risk in Communities (ARIC) study, the benefit of physical activity was previously shown to be independent of family history of premature coronary heart disease [26]. This finding is also in line with the results of this study, showing that also an overall favorable lifestyle (including physical activity) lowers the risk of CAD regardless of parental history of MI. Studies have shown that lifestyle counseling in the primary care for patients at high risk results in favorable lifestyle changes and lowers the risk of CAD [27–29]. Overall, our findings therefore reinforce that adherence to a favorable lifestyle is of considerable importance in the prevention of CAD [30], regardless of underlying baseline risk.

The strengths of this study include the large study population with minimal (< 0.5%) loss to follow-up due to linkage of national and regional registers with nearly complete coverage. In addition, this study is strengthened by the availability of extensive data from baseline examinations including dietary data of high relative validity [15, 31]. Our study has several limitations that should be discussed. Participants in the study joined spontaneously or were recruited mainly through invitations. It is possible that subjects who accepted the invitation differed from the ones who did not as only 40% of the eligible population joined the study. However, a previous study has shown that the socio-demographic structure, prevalence of smoking habits and obesity were similar compared to another survey in the same population [32]. Another limitation of this study is that diet and lifestyle were self-reported and all risk factors were assessed at baseline only. Both self-reported data and data based on assessment at a single point in time will be subject to measurement error. However, due to the prospective study design any exposure misclassification would most likely result in an attenuation of observed associations. Since this is an observational study there is always a possibility of residual confounding and there are several other putative factors contributing to the development of CVD, apart from the traditional risk factors considered in this study. Finally, the lifestyle score used in this study dichotomizes lifestyle exposures for simplicity in interpretation and assessment of adherence to current recommendations. The associations between the included lifestyle factors and risk of CAD are however likely to be continuous. Hence, the effect of the included individual lifestyle factors may be attenuated because of including subjects who may still derive a benefit at levels below our cut-points. Further, for assessment of lifestyle risk each lifestyle component contributed similarly to the overall score and no relative weighting of the individual components was performed.

## Conclusions

We observed that a favorable lifestyle is associated with around 40% reduced relative and 10-year cumulative risk of CAD across strata of non-modifiable risk factors, including age, gender, educational level and parental history of MI. These findings support the usefulness of lifestyle-targeted CAD prevention among subgroups at higher non-modifiable risk within the overall healthy population.

## Supplementary information

**Additional file 1: Table S1.** Classification of adherence to components of a healthy dietary pattern in the Malmö Diet and Cancer study. **Table S2.** Baseline characteristics by parental history of myocardial infarction in the Malmö Diet and Cancer Study (1991–1996). **Table S3.** Baseline characteristics by gender (male/female) in the Malmö Diet and Cancer Study (1991–1996). **Table S4.** Baseline characteristics by age groups in the Malmö Diet and Cancer Study (1991–1996). **Table S5.** Baseline characteristics by educational level in the Malmö Diet and Cancer Study (1991–1996). **Table S6.** Hazard ratios (95% confidence intervals) for incidence of coronary artery disease by lifestyle categories and non-modifiable risk factors. **Fig. S1.** Cumulative hazard of coronary events in the Malmö Diet and Cancer Study (1991–2014) by risk factor categories. Standardized event rates and hazard ratios (HRs; 95% confidence intervals) by lifestyle, age, gender, educational level, and parental history of MI are shown.

## Data Availability

The data analyzed in this study is available by application to the Malmö Diet and Cancer Study Steering Committee. The dataset analyzed is not publicly available due to restriction in the ethical permission. All codes and syntaxes used for analysis are available from the corresponding author upon reasonable request.

## Abbreviations

CAD: coronary artery disease
CABG: coronary artery bypass grafting
CI: confidence interval
CVD: cardiovascular disease
HR: hazard ratio
MDCS: Malmö Diet and Cancer Study
MI: myocardial infarction
PCI: percutaneous coronary intervention
