# Supplementary material for: A favorable lifestyle lowers the risk of coronary artery disease consistently across strata of non-modifiable risk factors in a population-based cohort
Source: BMC Public Health. 2019 Nov 27;19:1575. doi: 10.1186/s12889-019-7948-x (PMC6882082; doi:10.1186/s12889-019-7948-x)
Supplement: Supplementary file 1 — Additional file 1: Table S1. Classification of adherence to components of a healthy dietary pattern in the Malmö Diet and Cancer study. Table S2. Baseline characteristics by parental history of myocardial infarction in the Malmö Diet and Cancer Study (1991–1996). Table S3. Baseline characteristics by gender (male/female) in the Malmö Diet and Cancer Study (1991–1996). Table S4. Baseline characteristics by age groups in the Malmö Diet and Cancer Study (1991–1996). Table S5. Baseline characteristics by educational level in the Malmö Diet and Cancer Study (1991–1996). Table S6. Hazard ratios (95% confidence intervals) for incidence of coronary artery disease by lifestyle categories and non-modifiable risk factors. Fig. S1. Cumulative hazard of coronary events in the Malmö Diet and Cancer Study (1991–2014) by risk factor categories. Standardized event rates and hazard ratios (HRs; 95% confidence intervals) by lifestyle, age, gender, educational level, and parental history of MI are shown. [file 12889_2019_7948_MOESM1_ESM.docx]

**Supplemental material**

**A favorable lifestyle lowers the risk of coronary artery disease consistently across risk groups in a population-based cohort**

Kristian Dimovski^a^, Marju Orho-Melander^a^, Isabel Drake^a*^

^a^ Diabetes and cardiovascular disease – genetic epidemiology, Department of Clinical Sciences in Malmö, Lund University, Sweden

**Supplement Table 1**. Classification of adherence to components of a healthy dietary pattern in the in the Malmö Diet and Cancer study

|  |  | Cut-off | Serving size in gram | Included food items |
| --- | --- | --- | --- | --- |
| Consume more | Fruits | ≥ 3 servings/day | 100 g | All fruits, berries, and 100% fruit juice |
|  | Nuts, seeds | ≥ 1 serving/week | 28 g | All nuts |
|  | Vegetables | ≥ 3 servings/day | 75 g | All vegetables and legumes (not including potatoes)  ] |
|  | Whole grains | ≥ 3 servings/day | Whole-grain bread 35 g  Crisp bread 12 g  Biscuits 10 g  Cereals 40 g | High-fiber soft and crisp bread, high-fiber biscuits and rusks, high-fiber cereals |
|  | Fish | ≥ 2 servings/week | 125 g | Fish and shellfish |
|  | Dairy | ≥ 2.5 servings/day | Milk 250 g  Cheese 20 g  Yoghurt 250 g | Milk, cheese, fermented milk (yoghurt) |
| Consume less | Refined grains | ≤ 1.5 servings/day | Low-fiber bread 30 g  Cereals 40 g  Icecream 75 g  Cakes/cookies 40 g  Sweets 40 g  Sugar 13 g  Marmalade 20 g  Chocolate 40 g | Low-fiber bread and cereals, breakfast cereals with added sugar, sweets, cakes and biscuits, added sugar |
|  | Processed meats | ≤ 1 serving/week | 85 g | Preserved meats and processed meats |
|  | Unprocessed red meats | ≤ 1.5 servings/week | 100 g | Beef, pork, lamb (non-processed) |
|  | Sugar-sweetened beverages | ≤ 1 serving/week | 250 g | All sugar-sweetened beverages |

**Supplement Table 2**. Baseline characteristics by parental history of myocardial infarction in the Malmö Diet and Cancer Study (1991-1996).

|  | **No parental history of MI**  **N= 17896** | **Parental history of MI**  **N = 8427** | **P-value**** |
| --- | --- | --- | --- |
| **Age, years ^** | 58.1 (7.7) | 56.6 (7.5) | <0.0001 |
| **Gender ¤** |  |  | <0.0001 |
| **Male, %** | 40.0 | 34.2 |  |
| **Female, %** | 60.0 | 65.8 |  |
| **Education ¤** |  |  | <0.0001 |
| **Elementary, %** | 41.9 | 39.1 |  |
| **Secondary, %** | 35.1 | 36.4 |  |
| **University/college degree, %** | 23.1 | 24.5 |  |
| **ASCVD risk ≥7.5% *¤** | 50.2 | 46.8 | 0.025 |
| **History of Hypertension ¤** | 59.7 | 61.6 | 0.005 |
| **History of Diabetes Mellitus ¤** | 3.84 | 4.19 | 0.17 |
| **Body-mass Index, kg/m^2^ ^** | 25.7 (3.92) | 25.7 (3.95) | 0.947 |
| **Lipid Levels*** |  |  |  |
| **LDL Cholesterol, mg/dl ^** | 4.12 (0.99) | 4.27 (0.98) | <0.0001 |
| **HDL Cholesterol, mg/dl ^** | 1.39 (0.37) | 1.39 (0.38) | 0.47 |
| **Triglycerides, mg/dl #** | 1.25 (0.87–1.59) | 1.16 (0.87 – 1.61) | 0.96 |
| **Lipid-lowering Medication ¤** | 1.83 | 2.95 | <0.0001 |
| **C-Reactive Protein ***# | 1.3 (0.7 – 2.7) | 1.3 (0.6 – 2.7) | 0.58 |
| **Healthy lifestyle score ¤** |  |  | 0.12 |
| **Unfavorable** | 22.0 | 21.2 |  |
| **Intermediate** | 44.3 | 45.6 |  |
| **Favorable** | 33.7 | 33.3 |  |
| **HbA1C ***# | 4.8 (4.5 – 5.1) | 4.8 (4.5 – 5.1) | 0.53 |

* Only subjects from the MDC cardiovascular cohort (N=4,995).

^Continuous variables expressed as mean values with standard deviation in parenthesis. Differences in mean values were tested using one-way ANOVA.

#Skewed continuous values are expressed as median with interquartile range in parenthesis. Differences in median were tested using Kruskal-Willis ranksum test.

¤ The distribution of categorical variables are expressed as percentages of the total amount in each subgroup. The difference in distribution was tested using chi2 test.

****** P-values <0.05 were considered as statistically significant.

**Supplement Table 3**. Baseline characteristics by gender (male/female) in the Malmö Diet and Cancer Study (1991-1996).

|  | **Male**  **N= 10037** | **Female**  **N = 16286** | **P-value**** |
| --- | --- | --- | --- |
| **Age, years ^** | 59.0 (7.0) | 57.3 (7.9) | <0.0001 |
| **Education: ¤** |  |  | <0.0001 |
| **Elementary %** | 44,9 | 38.5 |  |
| **Secondary %** | 31.9 | 37.7 |  |
| **University/college degree, %** | 23.2 | 23.8 |  |
| **ASCVD risk ≥7.5% *¤** | 77.6 | 29.8 | <0.0001 |
| **History of Hypertension ¤** | 67.5 | 55.9 | <0.0001 |
| **History of Diabetes Mellitus ¤** | 50.7 | 49.3 | <0.0001 |
| **Body-mass Index, kg/m^2^ ^** | 26.2 (3.42) | 25.3 (4.18) | <0.0001 |
| **Lipid Levels*** |  |  |  |
| **LDL Cholesterol, mg/dl ^** | 4.13 (0.90) | 4.20 (1.04) | <0.0001 |
| **HDL Cholesterol, mg/dl ^** | 1.21 (0.30) | 1.51 (0.37) | <0.0001 |
| **Triglycerides, mg/dl #** | 1.28 (0.96 – 1.81) | 1.08 (0.81 – 1.48) | <0.0001 |
| **Lipid-lowering Medication ¤** | 3.0 | 1.7 | <0.0001 |
| **C-Reactive Protein *#** | 1.3 (0.7 – 2.7) | 1.3 (0.7 – 2.7) | 0.72 |
| **Healthy lifestyle score ¤** |  |  | <0.0001 |
| **Unfavorable** | 34.6 | 65.4 |  |
| **Intermediate** | 36.6 | 63.4 |  |
| **Favorable** | 42.4 | 57.6 |  |
| **Hba1c *#** | 4.8 (4.5 – 5.1) | 4.8 (4.5 – 5.1) | 0.23 |

* Only subjects from the MDC cardiovascular cohort (N=4,995).

^Continuous variables expressed as mean values with standard deviation in parenthesis. Differences in mean values were tested using one-way ANOVA.

#Skewed continuous values are expressed as median with interquartile range in parenthesis. Differences in median were tested using Kruskal-Wallis ranksum test.

¤ The distribution of categorical variables are expressed as percentages of the total amount in each subgroup. The difference in distribution was tested using chi2 test.

****** P-values <0.05 were considered as statistically significant.

**Supplement Table 4**. Baseline characteristics by age groups in the Malmö Diet and Cancer Study (1991-1996).

|  | **43.0-<54.9 years**  **N = 10740** | **55.0-64.9 years**  **N = 10117** | **65.0-74 years**  **N = 5466** | **P-value**** |
| --- | --- | --- | --- | --- |
| **Gender ¤** |  |  |  | <0.0001 |
| **Male, %** | 32.2 | 42.9 | 41.1 |  |
| **Female, %** | 67.8 | 57.2 | 58.9 |  |
| **Education ¤** |  |  |  | <0.0001 |
| **Elementary, %** | 28.8 | 47.1 | 53.4 |  |
| **Secondary, %** | 39.4 | 33.6 | 31.5 |  |
| **University/college degree, %** | 31.8 | 19.3 | 15.1 |  |
| **Family history of MI, % ¤** | 32.9 | 32.7 | 29.0 | <0.0001 |
| **ASCVD risk ≥7.5% *¤** | 23.1 | 57.7 | 91.2 | <0.0001 |
| **History of Hypertension ¤** | 44.6 | 65.5 | 81.7 | <0.0001 |
| **History of Diabetes Mellitus ¤** | 2.1 | 5.3 | 5.2 | <0.0001 |
| **Body-mass Index, kg/m^2^ ^** | 25.1 (3.86) | 26.0 (3.93) | 26.2 (3.85) | <0.0001 |
| **Lipid Levels*** |  |  |  |  |
| **LDL Cholesterol, mg/dl ^** | 3.97 (0.95) | 4.27 (0.99) | 4.35 (1.01) | <0.0001 |
| **HDL Cholesterol, mg/dl ^** | 1.38 (0.36) | 1.40 (0.38) | 1.35 (0.36) | 0.0078 |
| **Triglycerides, mg/dl #** | 1.09 (0.8 – 1.53) | 1.18 (0.89 – 1.61) | 1.28 (0.96 – 1.77) |  |
| **Lipid-lowering Medication ¤** | 1.0 | 2.9 | 3.3 | <0.0001 |
| **C-Reactive Protein* #** | 1.1 (0.6 – 2.3) | 1.4 (0.7 – 3.0) | 1.6 (0.8 – 3.0) | 0.0001 |
| **Hba1c *#** | 4.7 (4.4 – 5.0) | 4.8 (4.5 – 5.1) | 5.0 (4.7 – 5.3) | 0.0001 |

* Only subjects from the MDC cardiovascular cohort (N=4,995).

^Continuous variables expressed as mean values with standard deviation in parenthesis. Differences in mean values were tested using one-way ANOVA.

#Skewed continuous values are expressed as median with interquartile range in parenthesis. Differences in median were tested using Kruskal-Wallis ranksum test.

¤ The distribution of categorical variables are expressed as percentages of the total amount in each subgroup. The difference in distribution was tested using chi2 test.

****** P-values <0.05 were considered as statistically significant.

**Supplement Table 5**. Baseline characteristics by educational level in the Malmö Diet and Cancer Study (1991-1996).

|  | **Elementary**  **N = 10782** | **Secondary**  **N = 9346** | **University/college**  **N = 6195** | **P-value**** |
| --- | --- | --- | --- | --- |
| **Age, years ^** | 59.4 (7.2) | 57.2 (7.6) | 55.5 (7.4) | <0.0001 |
| **Gender ¤** |  |  |  | <0.0001 |
| **Male, %** | 41.8 | 34.3 | 37.5 |  |
| **Female, %** | 58.2 | 65.7 | 62.5 |  |
| **Family history of MI, % ¤** | 30.5 | 32.8 | 33.4 | <0.0001 |
| **ASCVD risk ≥7.5% ¤** | 56.9 | 44.1 | 39.9 | <0.0001 |
| **History of Hypertension ¤** | 67.9 | 58.8 | 49.4 | <0.0001 |
| **History of Diabetes Mellitus ¤** | 4.8 | 3.7 | 2.9 | <0.0001 |
| **Body-mass Index, kg/m^2^ ^** | 26.3 (4.02) | 25.4 (3.89) | 25.0 (3.67) | <0.0000 |
| **Lipid Levels*** |  |  |  |  |
| **LDL Cholesterol, mg/dl ^** | 4.23 (0.99) | 4.16 (0.98) | 4.06 (0.97) | <0.0001 |
| **HDL Cholesterol, mg/dl ^** | 1.35 (0.36) | 1.41 (0.38) | 1.44 (0.39) | <0.0001 |
| **Triglycerides, mg/dl #** | 1.21 (0.91 – 1.66) | 1.12 (0.84 – 1.55) | 1.09 (0.83 – 1.52) | 0.0001 |
| **Lipid-lowering Medication ¤** | 2.9 | 2.0 | 1.3 | <0.0001 |
| **C-Reactive Protein* #** | 1.5 (0.7 – 3.0) | 1.3 (0.6 – 2.6) | 1.1 (0.6 – 2.4) | 0.0001 |
| **Hba1c #** | 4.9 (4.5 – 5.2) | 4.8 (4.5 – 5.1) | 4.7 (4.5 – 5.0) | 0.0001 |

* Only subjects from the MDC cardiovascular cohort (N=4,995).

^Continuous variables expressed as mean values with standard deviation in parenthesis. Differences in mean values were tested using one-way ANOVA.

#Skewed continuous values are expressed as median with interquartile range in parenthesis. Differences in median were tested using Kruskal-Wallis ranksum test.

¤ The distribution of categorical variables are expressed as percentages of the total amount in each subgroup. The difference in distribution was tested using chi2 test.

****** P-values <0.05 were considered as statistically significant.


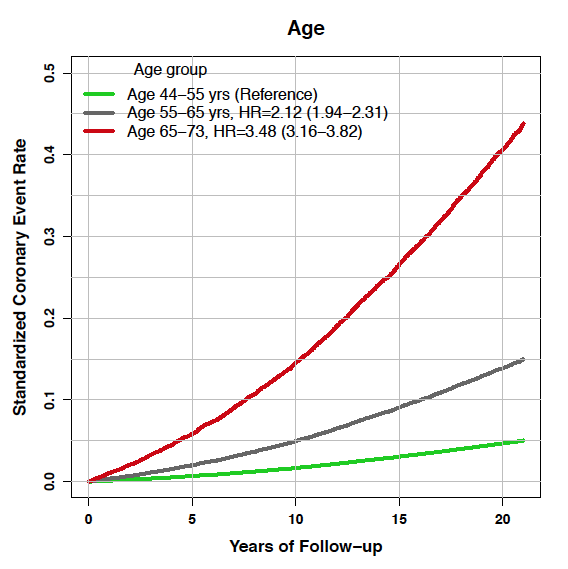

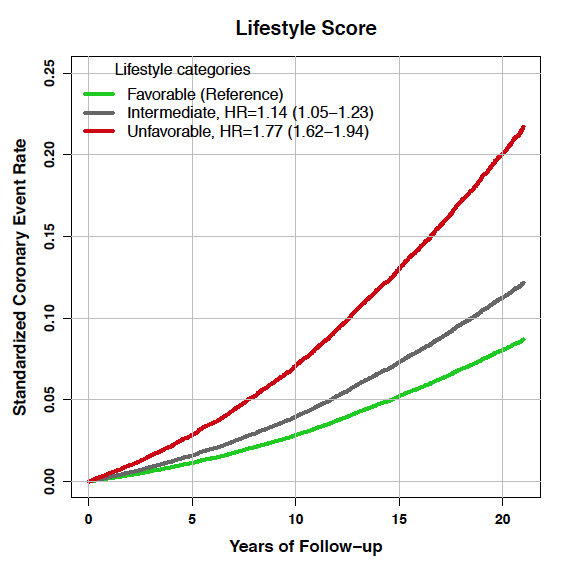

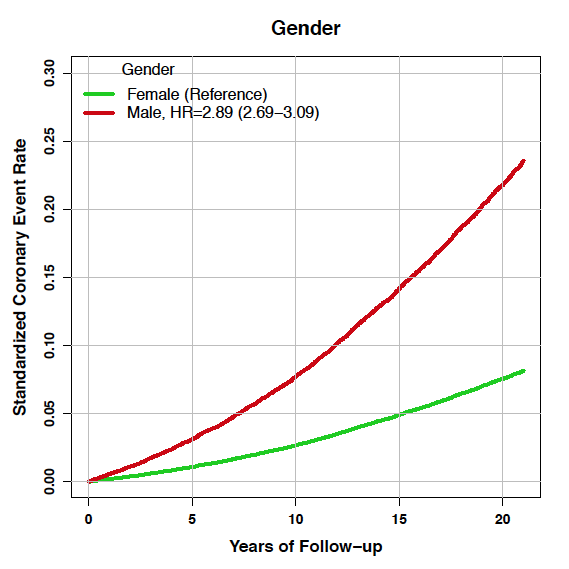

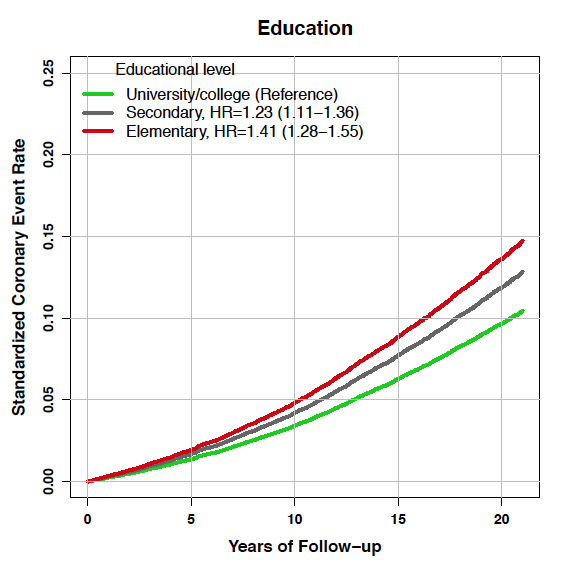

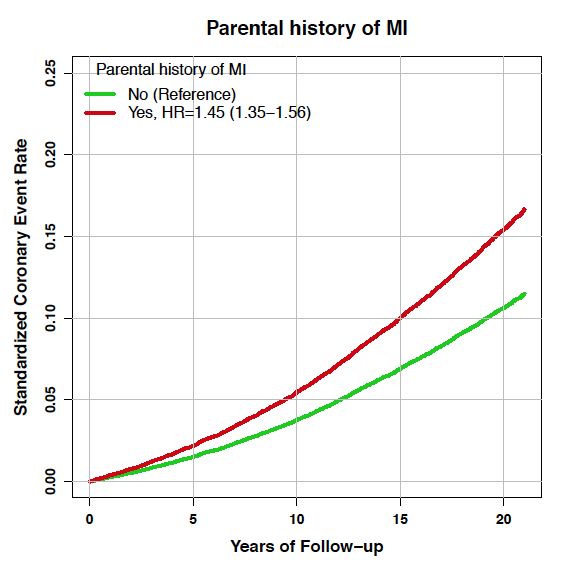


**Supplement Figure 1.** Cumulative hazard of coronary events in the Malmö Diet and Cancer Study (1991-2014) by risk factor categories. Standardized event rates and hazard ratios (HRs; 95% confidence intervals) by lifestyle, age, gender, educational level, and parental history of MI are shown.

**Supplement Table 6**. Hazard ratios (95% confidence intervals) for incidence of coronary artery disease by lifestyle categories and non-modifiable risk factors.

| Lifestyle categories | Risk groups | | |
| --- | --- | --- | --- |
|  | Age 65-73 | Age 55-65 | Age 43-55 |
| Unfavorable lifestyle | 1.00 (ref) | 0.58 (0.49 – 0.68) | 0.28 (0.23 – 0.33) |
| Intermediate lifestyle | 0.60 (0.51 – 0.70) | 0.38 (0.33 – 0.44) | 0.19 (0.16 – 0.23) |
| Favorable lifestyle | 0.57 (0.49 – 0.67) | 0.34 (0.29 – 0.40) | 0.14 (0.12 – 0.17) |
|  |  |  |  |
|  | Male | Female |  |
| Unfavorable lifestyle | 1.00 (ref) | 0.38 (0.33 – 0.43) |  |
| Intermediate lifestyle | 0.67 (0.60 – 0.75) | 0.23 (0.20 – 0.26) |  |
| Favorable lifestyle | 0.60 (0.54 – 0.68) | 0.19 (0.17 – 0.22) |  |
|  |  |  |  |
|  | Low education | Intermediate education | High education |
| Unfavorable lifestyle | 1.00 (ref) | 0.86 (0.74 – 0.99) | 0.69 (0.57 – 0.84) |
| Intermediate lifestyle | 0.64 (0.57 – 0.71) | 0.55 (0.48 – 0.62) | 0.47 (0.40 – 0.54) |
| Favorable lifestyle | 0.55 (0.49 – 0.63) | 0.50 (0.44 – 0.57) | 0.39 (0.33 – 0.46) |
|  |  |  |  |
|  | Parental history of MI | No parental history of MI |  |
| Unfavorable lifestyle | 1.00 (ref) | 0.67 (0.59 – 0.77) |  |
| Intermediate lifestyle | 0.63 (0.55 – 0.72) | 0.43 (0.38 – 0.49) |  |
| Favorable lifestyle | 0.55 (0.47 – 0.63) | 0.39 (0.34 – 0.44) |  |
|  |  |  |  |
